# The Diagnostic Value of the Added MR Imaging of the Scrotum in the Preoperative Workup of Sonographically Indeterminate Testicular Lesions—A Retrospective Multicenter Analysis

**DOI:** 10.3390/cancers14153594

**Published:** 2022-07-23

**Authors:** Susanne Deininger, Lukas Lusuardi, Maximilian Pallauf, Stefan Hecht, Rosemarie Forstner, Matthias Meissnitzer, Florian A. Distler, Eva Erne, Sebastian Graf, Sebastian Lenart, Juliane Putz, Christian Deininger, Peter Törzsök

**Affiliations:** 1 Department of Urology and Andrology, Paracelsus Medical University, 5020 Salzburg, Austria; s.deininger@salk.at (S.D.); l.lusuardi@salk.at (L.L.); m.pallauf@salk.at (M.P.); 2 Comprehensive Cancer Center, Medical University of Vienna, 1090 Vienna, Austria; 3 Department of Radiology, Paracelsus Medical University, 5020 Salzburg, Austria; s.hecht@salk.at (S.H.); r.forstner@salk.at (R.F.); m.meissnitzer@salk.at (M.M.); 4 Department of Urology, Paracelsus Medical University, 90419 Nürnberg, Germany; florian.distler@klinikum-nuernberg.de; 5 Department of Urology, Eberhard Karls University, 72076 Tübingen, Germany; eva.erne@med.uni-tuebingen.de; 6 Department of Urology and Andrology, Kepler University, 4020 Linz, Austria; sebastian.graf@kepleruniklinikum.at; 7 Department of Urology and Andrology, Barmherzige Brüder Hospital, 1020 Vienna, Austria; sebastian.lenart@gmx.at; 8 Department of Urology, Carl Gustav Carus University, Technische Universität Dresden, 01307 Dresden, Germany; juliane.putz@uniklinikum-dresden.de; 9 Institute of Tendon and Bone Regeneration, Spinal Cord Injury & Tissue Regeneration Center Salzburg, 5020 Salzburg, Austria; c.deininger@salk.at

**Keywords:** testicular tumor, cancer, seminoma, NSGCT, MRI, imaging, ultrasound

## Abstract

**Simple Summary:**

This retrospective multi-center study analyzes the diagnostic accuracy of magnetic resonance imaging (MRI) of the scrotum in comparison with standard ultrasound (US) and histopathological specimens. A total of *N* = 113 patients were included. A total of 53 histopathological specimens were available, with 52.8% malignant and 50.9% benign findings. Related to the histopathology, imaging was true negative, false negative, false positive and true positive in 4.1%, 2.1%, 25.0% and 37.5% for standard ultrasound (US) and 9.1%, 1.8%, 25.5% and 43.6% for MRI. Sensitivity, specificity, positive predictive value (PPV) and negative predictive value (NPV) were 94.7%, 20.0%, 36.0% and 88.9% for US and 85.7%, 72.8%, 52.1% and 93.7% for MRI, respectively. Benign lesions were significantly smaller than malignant ones on standard US, MRI and histopathology (all *p* < 0.05).

**Abstract:**

Background: The purpose of this study was to retrospectively analyze the diagnostic accuracy of magnetic resonance imaging (MRI) examinations of the scrotum in comparison with standard ultrasound (US) and histopathology. Methods: A retrospective multi-center analysis of MRI examinations of the scrotum performed between 06/2008 and 04/2021 was conducted. Results: A total of *n* = 113 patients were included. A total of 53 histopathologies were available, with 52.8% malignant and 50.9% benign findings. Related to histopathology, imaging was true negative, false negative, false positive, and true positive in 4.1%, 2.1%, 25.0% and 37.5% for standard ultrasound (US) and 9.1%, 1.8%, 25.5% and 43.6% for MRI. Sensitivity, specificity, positive predictive value and negative predictive value were 94.7%, 20.0%, 36.0% and 88.9% for US and 85.7%, 72.8%, 52.1% and 93.7% for MRI, respectively. Benign lesions were significantly smaller than malignant ones in standard US (*p* = 0.001), histopathology (*p* = 0.001) and MRI (*p* = 0.004). The size of malignant tumors did not differ significantly between histopathology and standard US (0.72) and between histopathology and MRI (*p* = 0.88). Conclusions: MRI shows good sensitivity and specificity for the estimation of testicular tumors in this collective. Benign lesions are significantly smaller than malignant ones. Both MRI and US can estimate the size of malignant tumors adequately.

## 1. Introduction

Testicular tumors account for 25% of malignant tumors in men between 20 and 40 years of age and present the most common malignancies in this age group. Overall, these tumors are rare, accounting for 1–2% of all adult neoplasms. The lifetime risk is approximately 0.4% [1], but the incidence has been increasing in recent years [2]. A total of 95% of all testicular tumors are malignant, with germ cell tumors (GCTs) being the most frequent histopathology [3]. The most common benign tumor is the Leydig cell tumor (LCT), followed by the Sertoli cell tumor, adenomatoid tumor, pseudofibrotic tumor of the tunica albuginea, epidermoid cyst and tubular fibrosis [4].

Some of the testicular lesions are palpable, and others are detected incidentally, e.g., during urological or andrological examinations. Up to 80% of non-palpable testicular lesions are benign [5,6], whereas palpable masses tend to be malignant (around 90%) [7]. Whether palpable or not, the diagnosis is mostly made by ultrasound (US) of the testis. The European Association of Urology (EAU) guidelines for testicular cancer [8] recommend the use of high-frequency (>10 Megahertz) ultrasound probes in testicular US to determine both location (intra- or extra-testicular) and size of the lesion. The examination of the contralateral testis is also essential to exclude lesions or risk factors for germ cell neoplasia in situ (GCNIS), such as microlithiasis.

Particularly in the case of small lesions, a reliable classification by US alone can be difficult. Until histopathology is obtained via surgery, the final diagnosis usually remains indeterminate. According to the EAU guideline, organ-preserving surgery with an intraoperative fresh frozen section (FFS) procedure is an option for indeterminate masses in singular testes, bilateral lesions or small, non-palpable lesions [8]. However, organ-preserving tumor enucleation is only feasible up to a certain tumor volume and only in certain locations in the testis. Nevertheless, also larger tumors can be benign. If the distinction between benign and malignant lesions is possible based on imaging with acceptable diagnostic accuracy, even more lesions can be treated conservatively.

The role of complementary MRI in the primary diagnosis remains that of a problem-solving tool, e.g., the EAU guideline only recommends MRI for the differentiation of intra- and extratesticular lesions [6,8,9,10,11,12].

However, MRI has not yet been able to develop its full competence in testicular tumor diagnosis. The aim of the study is a retrospective assessment of the performance of conventional ultrasound (US) and MRI examinations of the testis in six major urological departments. Subsequently, histopathological correlation of the surgical specimen was used to determine the sensitivity and specificity of US and MRI in the diagnosis of testicular lesions, focusing specially on the potential benefit of adding MRI of the scrotum.

## 2. Materials and Methods

### 2.1. Data Collection

The consent of the ethics committee of the Province of Salzburg was obtained (registration number 1194/2020), and the study was conducted in accordance with the Declaration of Helsinki. A retrospective analysis of the data of patients who received an MRI examination of the scrotum at six major urological departments in Austria and Germany (University Clinic of Urology and Andrology Salzburg, University Clinic of Urology Nürnberg, Department of Urology, University Hospital Carl Gustav Carus Dresden, University Clinic of Urology and Andrology Linz, Department of Urology and Andrology, Barmherzige Brüder Hospital Wien and University Clinic of Urology Tübingen) between June 2008 and April 2021 was conducted.

The following inclusion criteria were applied: complete patient and diagnostic data available, MRI of the scrotum performed in the predefined period at one of the study centers with the indication “indeterminate findings of an US of the testis” and aged between 18 and 99 years at the time of the MRI.

The following patient characteristics were collected using the respective clinic’s internal data programs: age, medical history (history of smoking, testicular tumor, undescended testis or testicular/scrotal operations and the presence of symptoms), the type of diagnosis, findings by physical examination (patient height and weight, Body Mass Index [BMI], laboratory values preoperatively (tumor markers alpha-fetoprotein [AFP], human chorionic gonadotropin [βHCG], lactate dehydrogenase [LDH] and placental alkaline phosphatase [PLAP]; sexual hormones follicle-stimulating hormone [FSH], luteinizing hormone [LH] and testosterone at the time of primary diagnosis), data on the surgery if performed (time from the first presentation to operation, duration, surgical access, the type of operation and fresh frozen section [FFS] procedure) and on the final histopathology (malignant or benign histopathology, tumor size, anatomical tumor position, T-status, in seminoma presence of rete testis infiltration, the presence of concomitant testicular intraepithelial neoplasia [TIN] and the consistency of FFS with final histology) and the following therapy of the patient.

The following imaging parameters and findings were analyzed based on the original reports: US findings (tumor site, tumor size and volume of testis), MRI data and findings (indication, technical details [Tesla, manufacturer and sequences], tumor site, tumor size and volume of testis). A repeated analysis of the images was not performed.

### 2.2. MRI Criteria for the Characterization of Malignant Tumors

The MRI criteria used to characterize malignant testicular tumors were based on previously published studies [13,14,15].

The presence of a multinodular/lobulated intratesticular lesion of mainly low signal intensity on T2-weighted images or an inhomogeneous lesion with heterogeneous signal intensity on T2-weighted images was considered suggestive of malignancy. Contrast enhancement, especially if heterogeneous, also was considered indicative of malignancy. The presence of a haemorrhage and/or the presence of necrosis and the extension of the neoplasm to the testicular tunicae, epididymis or spermatic cord was considered highly indicative of malignancy.

DWI images and apparent diffusion coefficient (ADC) maps were (when available) visually evaluated in order to assess the signal intensity of the lesions. Diffusion was considered restricted in the case of hyperintensity on high b-value DWI sequences (b = 1000 s/mm^2^) and hypointensity on ADC maps according to Bakir et al. [16]. Restricted diffusion was considered indicative of malignancy.

The absence of the above-mentioned MR imaging characteristics was considered indicative of benign testicular tumors.

### 2.3. Terminology

If histopathology is considered as the gold standard in this analysis, the findings of US and MRI can be classified accordingly. In the following analysis, the finding of a malignant tumor in histology is used as “positive”, and that of a benign tumor in histopathology or imaging is “negative”. The terms are defined as follows:

- False positive: incorrect detection of malignant finding via US/MRI despite the presence of a benign finding.
- True positive: correct detection of malignancy via US/MRI.
- False negative: incorrect detection of a benign finding via US/MRI despite the presence of malignancy.
- True negative: correct detection of a benign finding via US/MRI.

### 2.4. Statistics

#### 2.4.1. Comparisons of Two Subgroups

All data of continuous variables were checked for normal distribution (test of normality: Kolmogorov–Smirnov with Lilliefors significance correction, type I error = 10%) and with normal distribution also for heteroscedasticity (Levene test, type I error = 5%). In the case of normality and variance homogeneity, an independent two-sample t-test was used for group comparisons. In the case of normality but with no variance homogeneity, Welch’s *t*-test was applied. For variables without normally distributed data and for variables measured on ordinal scales, the exact Mann–Whitney U-test was used. Dichotomous variables were compared by Fisher’s exact test, and the other categorical variables were compared by the exact chi-squared test.

#### 2.4.2. Comparisons of More Than Two Subgroups

Comparisons of continuous variables with normally distributed data and variance homogeneity were performed by a parametric analysis of variance (ANOVA; post hoc tests by Hochberg’s GT2 method). For comparisons of all other continuous variables and of variables measured on ordinal scales, a non-parametric analysis of variance (Kruskal–Wallis test, followed by Nemenyi’s multiple comparisons) was used. Data of categorical variables were compared by the chi-squared test (exact or with Monte Carlo simulation, with the provision of adjusted residuals).

The influence of patient age, history of testicular tumors, presence of symptoms, US findings and correct diagnosis on MRI was investigated by logistic regression analyses. Another logistic regression analysis was used to investigate the predictability of a malignant tumor on MRI plus the covariates mentioned above.

Two-sided 95% confidence intervals (CI) were calculated according to the nature of the data (parametric, non-parametric or according to Clopper–Pearson).

The type I error was not adjusted for multiple testing. Therefore, the results of inferential statistics are descriptive only. Statistical analyses were performed using the open-source R statistical software package, version 4.0.5 (The R Foundation for Statistical Computing, Vienna, Austria). The detailed statistical analysis can be obtained on request from the authors.

## 3. Results

### 3.1. Epidemiology

A total of 116 MRI examinations in the predefined time period were identified, but *n* = 3 were excluded (one patient did not meet the final inclusion criterion “age between 18 and 99 years”, and two patients received 2 MRI examinations each). Therefore, finally *n* = 113 patients could be included in the statistical analysis. The following study centers participated and included the following number of patients: University Clinic of Urology and Andrology Salzburg (*n* = 58), University Clinic of Urology Nürnberg (*n* = 23), Department of Urology, University Hospital Carl Gustav Carus Dresden (*n* = 10), University Clinic of Urology and Andrology Linz (*n* = 11), Department of Urology and Andrology, Barmherzige Brüder Hospital Wien (*n* = 6) and University Clinic of Urology Tübingen (*n* = 5).

The median age of the patients was 39.0 years (range 16–89 years). A total of 10 patients were active smokers. In *n* = 19 patients, the medical history was positive for testicular tumors. In these cases, no previous systemic therapy had been required in *n* = 8, whereas *n* = 9 had received previous platinum containing chemotherapy and *n* = 2 radiotherapy. A total of 6 patients had a history of undescended testis. A total of 68 patients reported symptoms at initial presentation, including a palpable scrotal mass in *n* = 31, pain in *n* = 30 and infection in *n* = 5. The suspicious scrotal finding was incidental in *n* = 45, and the finding was detected during self-examination in *n* = 34 or during follow-up in *n* = 25. The tumor was located on the left side in *n* = 53, on the right side in *n*= 48 and on both sides in *n* = 5. The median volume of the affected testis in MRI or US was 14.0 mL (range 2.0–45.0 mL). The median tumor size in US was 0.6 cm (range 0–3.0 cm) for benign and 1.4 cm (range 0.5–15.0 cm) for malignant tumors.

A total of 8 patients had positive tumor markers, and *n* = 3 showed testosterone deficiency.

Indications for MRI were indeterminate sonographic findings (*n* = 98), the elevation of tumor markers or the exclusion of testicular primaries (*n* = 5) and pain (*n* = 3). As a result of the retrospective and multicentric study design, MR protocols were not standardized regarding field strength and sequences. All examinations were performed on either a 1.5 (*n* = 24) or 3 T (*n* = 82) Phillips (Philips Healthcare, Best, The Netherlands) or Siemens (Siemens Healthcare, Erlangen, Germany) scanners. Most examinations (*n* = 57) included, in addition to standard T1 and T2 weighted sequences, contrast-enhanced sequences and diffusion-weighted sequences (DWI), and some included contrast-enhanced sequences but no DWI sequences (*n* = 40). Moreover, *n* = 2 examinations were performed without contrast but with DWI sequences. The median tumor size in MRI was 0.8 cm (range 0.1–14.0 cm) for benign and 1.7 cm (range 0.5–15.0 cm) for malignant tumors. MRI revealed benign, malignant and indeterminate findings in *n* = 60, *n* = 38 and *n* = 11 cases, respectively.

**Figure 1:**
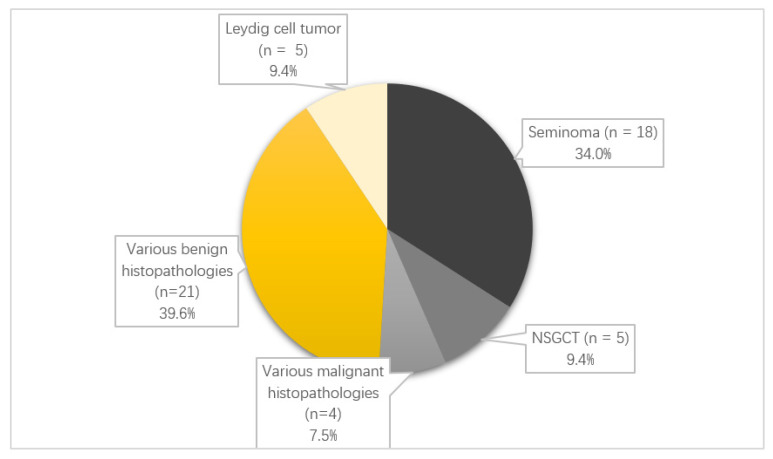
Distribution of histopathology (*n* = 53; various malignant histopathologies: adenocarcinoma of the rete testis, liposarcoma of the spermatic cord, myeloid sarcoma of the testis and pancreatic analogous solid-pseudopapillary neoplasm of the testis; various benign histopathologies: infection (*n* = 4), residual fibrosis after chemotherapeutically pretreated teratoma (*n* = 1), normal testicular tissue (*n* = 5), sclerosis/fibrosis (*n* = 2), Sertoli cell hyperplasia/tumor (*n* = 2), Leydig cell hyperplasia (*n* = 1), atrophy (*n* = 4), hematoma (*n* = 1) and St. p. testicular torsion (*n* = 1)).

A surgical intervention was performed in *n* = 55 patients, including *n* = 33 orchiectomies (*n* = 10 with benign and *n* = 23 with malignant findings), and *n* = 20 partial testicular resections or biopsies (*n* = 16 with benign and *n* = 4 with malignant findings). In *n* = 24 patients, a fresh frozen section (FFS) was performed during surgery. Due to the definite exclusion of malignant dignity in MRI (e.g., varicocele), *n* = 58 patients did not undergo surgical intervention. A total of 28 cases showed benign findings in histopathology, whereas *n* = 27 were malignant. However, *n* = 2 histopathologies were not primary testicular or epididymal tumors and were only located intrascrotally, which is why they were excluded from statistical analyses. The distribution of histopathologies for the *n* = 53 specimens can be found in Figure 1. Median tumor size was 0.6 cm (range 0.3–1.0 cm) for benign and 1.7 cm (range 0.1–15.0 cm) for malignant tumors. In *n* = 23 cases, FFS and the final histopathology were concordant.

### 3.2. The Rate of True Positive, False Positive, True Negative and False Negative US/MRI Findings

A total number of *n* = 48 (for US) and *n* = 55 (for MRI) histopathological specimens with concurrent preoperative imaging findings were available for this analysis. The rate of true negative, false negative, false positive, true positive and indeterminate US/MRI findings regarding final histopathology results can be found in Table 1.

### 3.3. Sensitivity, Specificity, Positive Predictive Value (PPV) and Negative Predictive Value (NPV) of US and MRI

In reference to final histopathology sensitivity, the specificity, PPV and NPV for the detection of benign and malignant tumors for US and MRI were calculated and are summarized in Table 2. Patients with unequivocally benign findings in MRI did not undergo surgery and were therefore automatically assigned to the “true negative” findings for this calculation.

### 3.4. Subgroup of Positive Tumor Marker Patients

Among study participants, there were *n* = 8 cases with positive tumor markers. The individual cases can be found in Table 3.

At a significance level of *p* < 0.05, there was no significant correlation between the parameter “positive tumor marker” and the correctness of MRI findings in our collective. The statistical analysis revealed a *p*-value of 0.086.

### 3.5. Subgroup Comparison: Benign Versus Malignant Histopathology

**Figure 2:**
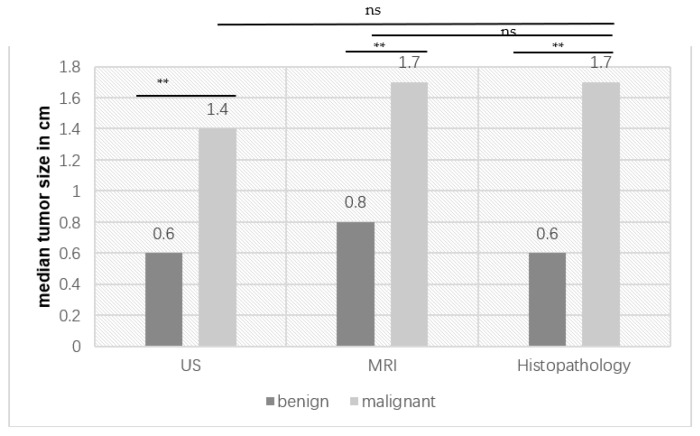
Median tumor size of benign and malignant tumors in ultrasound (US), magnetic resonance imaging (MRI) and histopathology (** *p* < 0.001) (ns = not significant).

The clinical and diagnostic parameters were compared between the subgroups of patients with benign and malignant histopathology, and the results are given in Table 4 and Figure 2. Only a selected presentation of the most important clinical parameters is given, and further data can be obtained on request from the authors.

The median tumor sizes for benign and malignant tumors in US, MRI and histopathology are listed in Figure 2. Lesion size was significantly different for benign and malignant tumors in all three modalities. There was no significant difference in size between US and histopathology (*p* = 0.7214) and between MRI and histopathology (*p* = 0.8822) (unpaired *t*-test with Welch’s correction).

### 3.6. Analysis of the Performance of the Frozen Section (FFS) Examination

In addition, the performance of the FFS and the consistency with the final histology regarding benign/malignant findings were analyzed. A total number of *n* = 24 FFS examinations were performed in a total number of *n* = 53 available final testicular or epididymal histopathological specimens. The FFS correlated with the final histopathology in 92.0% of cases. FFS was false positive in 0%, true positive in 40.0%, false negative in 4.0%, true negative in 48.0% and indeterminate in 8.0%. Overall, FFS was correct in 88.0% of cases and incorrect in 4.0% of cases.

## 4. Discussion

For decades, US has been the standard diagnostic tool in the diagnosis and workup of testicular tumors, mostly due to low costs and wide availability in outpatient urological care [17]. However, MRI of the scrotum as a second-line diagnostic tool has the potential to improve patient care and reduce the number of unnecessary surgical procedures [18]. The use of standard US in the diagnosis of malignant testicular tumors shows a sensitivity of 92–98% with a specificity of 95–99.8% in the literature [19], which seems surprisingly high compared to our data. Here, the sensitivity of the standard US examination is comparable with 94.7%, and the specificity with only 20.0% is clearly lower. One possible explanation for this is the fact that US is highly examiner-dependent, and with US examinations performed in six different clinics, numerous physicians with different experience levels were involved. No standardized protocol for the US and for its documentation was used. However, the addition of further US modalities such as contrast media (CM)-enhanced US or real-time or shear wave elastography has been shown to further improve the diagnostic value of US in this field and to help to differentiate between malignant and benign findings. Isidori et al. demonstrated a high accuracy for the combination of gray scale and CM-enhanced US (area under the ROC receiver operating characteristic curve performance: 0.927) in 2014 [20]. Reginelli et al. showed in 2019 that the addition of real-time elastography to gray-scale and Doppler US resulted in a sensitivity of 100%, a specificity of 83%, an NPV of 100% and a PPV of 91% in the diagnosis of malignant testicular tumors [21].

The data for US is quite heterogeneous, and the expectation is for MRI to provide additional information, especially in indeterminate testicular lesions. MRI of the scrotum demonstrates generally high sensitivity (up to 100%) and specificity (up to 88%) in the differentiation of benign and malignant lesions [13]. In our study, sensitivity with 85.7% and specificity with 72.8% were also relatively high. The advantage of MRI compared to gray scale US is obvious: the use of multiparametric MR protocols, including diffusion-weighted imaging and contrast-enhanced sequences, should add information in lesion characterization. In the literature, using advanced MRI techniques with different dignities of tumors revealed different contrast media enhancement. Benign testicular tumors, such as LCT, demonstrated higher maximal relative contrast enhancement and shorter time to maximal enhancement compared to seminomas in some studies [10,22], whereas malignant lesions showed a lower apparent diffusion coefficient using DWI [23]. In a retrospective study of 44 men, three types of time-signal intensity curves were defined [11]: linear increasing accumulation, typical for healthy tissue (type 1 curve); rapid increase in enhancement followed by plateauing or a smaller increase in enhancement, typical of benign lesions (type 2 curve); and rapid increase in enhancement followed by steady washout, typical of malignant lesions (type 3 curve). Wang et al. 2021 showed that, in the diagnosis of indeterminate testicular lesions, the specificity and accuracy increased by 9.8% and 3.2%, respectively, when adding DWI to conventional MRI [24]. Particularly in the distinction between seminoma, NSGCT and lymphoma cystic changes, T2-hypointensity, intratumoral septa and ADC value were independent factors.

Due to the heterogeneous data situation, with different MRI protocols, it was not possible within the framework of our study to determine the ideal MRI protocol for lesion characterization. To address this question, and to perform a high-quality comparison of gray scale US, CM-enhanced US, real-time elastography and MRI with a histopathological correlation, our research group is currently conducting a prospective study of patients with testicular tumors who are already scheduled for surgery.

An interesting result of our study is that benign tumors are significantly smaller than malignant tumors in all three techniques (US (*p* = 0.001), histopathology (*p* = 0.001) and MRI (*p* = 0.004). Our findings are correlated with the literature. Shilo et al. showed in 2012 that, in their study cohort of 131 patients with testicular tumors, benign lesions were 63.4% smaller than malignant ones (1.5 vs. 4.1 cm). It is interesting to note that the study group calculated a cut-off value of 1.9 cm, above which the percentage of benign findings was only 2%, below the percentage was 38.5% (*p* < 0.05) [25]. In our cohort, the median size of malignant lesions was only 1.4–1.7 cm, and the median size of benign lesions was 0.6–0.8 cm, which is thus clearly smaller than even the cut-off value of the research group of Shilo et al. In our cohort of malignant lesions, it was not possible to calculate a sharp cut-off.

As the lesion becomes larger, it becomes more likely to be palpable, and according to previous data, 90–95% of all palpable tumors of the testis are malignant [26]. At primary diagnosis, malignant testicular tumors have a median size of 3 cm (IQR 1.8–4.5 cm) [27]. Abboudi et al. also showed in 2013 that over two-thirds of testicular tumors < 1 cm were benign [28].

Nevertheless, the size of a lesion cannot provide reliable information of its dignity, and with smaller sizes, US and MRI assessment is more challenging. Bieniek et al. showed in 2018 a rate of indeterminate testicular lesions diagnosed by US smaller than 1 cm of 2.9% in a population of >4000 men undergoing urological treatment for fertility evaluation. Of these patients, *n* = 18 underwent histopathological confirmation, and *n* = 6 were found to have a malignancy. However, these cases showed no significant differences in size and vascularity on preoperative US examination compared with the benign lesions [29].

As a testicular lesion becomes smaller, it becomes more likely that organ-preserving surgery (testis-preserving surgery (TSS)) can be performed. The German Testicular Cancer Study Group (GTCSG) proposed in 2006 the option of TSS in the case of the following parameters: a tumor in a solitary testis or bilateral tumors, diameter < 2 cm, no invasion of the rete testis, biopsies of the surgical bed, normal preoperative LH and testosterone levels and good patient compliance [30].

Especially in the planning of TSS, the preoperative estimation of the size of the lesion is crucial. In the standard preoperative preparation, this is performed with US. Assuming that the tumor size from histopathology is the one most likely to be correct, we could not reveal any significant difference in the histopathologic correlation between the sizes of malignant lesions in US and in MRI. This means that both examination methods can already estimate the size of a lesion very well. Other research demonstrated a tendency to underestimate the size of a testicular lesion by US examination [31], which could be another advantage of MRI.

Among our study participants, there were *n* = 8 patients with positive tumor markers. At first glance, one may think that this would speak against the intended question of this manuscript concerning the use of MRI in the diagnosis of unclear testicular lesions. However, the indication for MRI in these patients was partly to search for tumors with unclearly elevated tumor markers or to search for possible primaries in extratesticular (e.g., mediastinal) germ cell tumors. Other manuscripts with similar research questions do not mention specific tumor markers [32]. For the sake of completeness, and because of the importance we attach to this laboratory chemical finding in diagnostics, we decided to mention the tumor markers. In addition, there was no statistically significant correlation between positive tumor markers and the correctness of the MRI in the diagnosis of indeterminate testicular lesions (*p* > 0.05) in our cohort.

Another important point in the planning, particularly of a one-step TSS, is the application of FFS. The intraoperative diagnosis of a benign tumor in the FFS, which was subsequently revealed to be a malignant tumor in the final histopathology (=false negative), would be a disadvantage for any patient because of the more restrained resection with a benign preliminary finding. In our study, the FFS showed a convincing concordance of 88.0% with the final histopathology, with only 4.0% of false negative findings. The literature also shows concordance rates of up to 100% between FFS and final histopathology [33]. It is important to notice that burned-out tumors can be diagnosed as fibrosis in histopathology; thus, a benign finding can reflect a malignant process as well [22]. In our study, only two patients were diagnosed with fibrosis: one of the patients had a residual fibrosis after chemotherapy, and one patient did not have a testicular tumor in the medical history.

Nevertheless, this study has some limitations. Due to the retrospective and multi-center design, some mainly anamnestic data are available from only a few patients. For this reason, some statistical evaluations based on these data are certainly of limited value. Patients with clearly benign findings (e.g., varicoceles or similar findings) in MRI were often not surgically explored. Therefore, a reliable histological diagnosis is missing. Even if a benign dignity can be assumed, malignancy cannot be excluded with certainty in every case. In addition, the number of histopathologies is relatively low, with *n* = 53/55. Due to the partially small amount of data, statistical differentiation between tumors of testis, epididymis and spermatic cord was also omitted, which certainly favors inaccuracy in the evaluation. The heterogeneity of the examinations at six different clinics because of the lack of established protocols for the examination of the testis (US and MRI) limits the accessibility of the overall cohort. To our knowledge, this study represents one of the largest investigations of MRI examinations of the scrotum. Due to the low incidence of testicular tumors in the general population (year 2017 incidence in Austria *n* = 438 [34]), the comparatively high costs of MRI and the standard surgical procedure for suspected testicular cancer, high-quality studies with large cohorts are difficult to realize for our research question. Wang et al. included *n* = 63 patients with MRI of the scrotum and associated histopathology in their study in 2021 [24], and Patel et al. [23] published about *n* = 217 such cases, including nine different studies, in a systemic review in 2020. Moreover, the still-widespread clinical approach of rapid surgical treatment of such patients and long waiting times for MRI examinations often argue against such imaging diagnostics. Especially in relation to the simultaneously available US and histopathology studies, it still has a significance in the exploration of the diagnostic value of MRI in this indication. To obtain higher quality data, a prospective clinical study with the same research question is currently taking place at Salzburg University Clinic.

## 5. Conclusions

In our study, benign testicular lesions were significantly smaller than malignant tumors. This would allow for TSS in many cases. MRI shows good performance of sensitivity and specificity in the differentiation between malignant and benign lesions and good prediction of the size of the tumors. US is more sensitive but much less specific in detecting tumors of the testis. MRI of the testis could, therefore, be suitable in special cases for decision making and better counseling of the patient. Prospective trials must address optimal indications, as well as technical details for MRI examinations, to further improve the diagnosis of indeterminate testicular lesions. The long-term goal would be to dispense surgical exposure and histopathological confirmation in unequivocally benign findings in the future.

## Figures and Tables

**Table 1:** Rate of true negative, false negative, false positive, true positive and indeterminate preoperative ultrasound (US) and magnetic resonance imaging (MRI) findings regarding histopathology results.

		US Finding				MRI Finding			
		*n* Benign (%)	*n* Malignant (%)	*n* Indeterminate (%)		*n* Benign (%)	*n* Malignant (%)	*n* Indeterminate (%)	
Histology	*n* benign (%)	2 (4.1)	12 (25.0)	11 (22.9)	25 (52.1)	5 (9.1)	14 (25.5)	8 (14.5)	27 (49.1)
	*n* malignant (%)	1 (2.1)	18 (37.5)	4 (8.3)	23 (47.9)	1 (1.8)	24 (43.6)	3 (5.5)	28 (50.9)
		3 (6.2)	30 (62.5)	15 (31.3)	48 (100)	6 (10.9)	38 (69.1)	11 (20.0)	55 (100)

**Table 2:** Sensitivity, specificity, positive predictive value (PPV) and negative predictive value (NPV) of ultrasound (US) and magnetic resonance imaging (MRI) for the detection of benign and malignant testicular tumors (CI = Confidence Interval). For statistical analysis of MRI data, the category “indeterminate” was assigned to the opposite gold standard category in the sense of a worst-case approach.

	US	MRI
Sensitivity in % (95%-CI)	94.7 (74.0–99.9)	85.7 (67.3–96.0)
Specificity in % (95%-CI)	20.0 (9.1–35.7)	72.8 (61.8–82.1)
PPV in % (95%-CI)	36.0 (22.9–50.8)	52.1 (37.0–67.1)
NPV I % (95%-CI)	88.9 (51.8–99.7)	93.7 (84.5–98.2)

**Table 3:** Positive tumor marker patients included in the study (*n* = 8; MRI: magnetic resonance imaging; AFP: alpha-fetoprotein; LDH: lactate dehydrogenase; HCG: human chorionic gonadotrophin).

Patient Nr.	LDH (135–225 U/L)	HCG (<1 U/L)	AFP (0.5–10 µg/L)	Indication for MRI
1	150	18	2.2	Exclusion of testicular lesion in the case of cervical lymphadenopathy (Choriocarinoma)
2	162	<1	12.9	Unclear increase in AFP
3	190	<1	79.2	Indeterminate testicular lesion
4	NA	25	47.5	Indeterminate testicular lesion, mental retardation
5	178	4	14.3	Lymphadenopathy; MRI was performed during upfront chemotherapy for metastatic tumors
6	379	>2.0	2465	Mediastinal primum; search for the origin
7	254	11.67	4.3	Indeterminate testicular lesion
8	115	<2.3	10.85	Indeterminate testicular lesion

**Table 4:** Selected *p*-values comparing clinical and diagnostic parameters of the two subgroups of patients with benign and malignant histopathology (* *p* < 0.05, ** *p* < 0.01).

Parameter	*p*-Value
Age	0.207
History of smoking	0.161
History of testicular tumors	0.252
History of undescended testis	0.661
Presence of symptoms	0.128
Median volume of the affected testicle in MRI or ultrasound	0.676
Tumor size for US	0.001 **
Tumor size in histopathology	0.001 **
Positive tumor markers (AFP, βHCG, HPLAP, LDH)	0.014 *
Presence of testosterone deficiency	0.541
Tumor size in MRI	0.004 **

## Data Availability

The complete data with statistical analyses can be obtained on request from the authors.

## Abbreviations

AFP	alpha-fetoprotein
BMI	Body Mass Index
CM	Contrast median
CT	Chemotherapy
DWI	Diffusion-Weighted Imaging
EAU	European Association of Urology
FFS	Fresh frozen section
FS	Fatsat
FSH	Follicle-stimulating hormone
GCNIS	Germ cell neoplasia in situ
HPLAP	Human placental alkaline phosphatase
LCT	Leydig cell tumor
LDH	Lactate dehydrogenase
LH	Luteinizing hormone
MRI	Magnetic resonance imaging
NPV	Negative predictive value
ns	Not significant
NSGCT	Nonseminomatous germ cell tumor
PD	Proton-density weighted
PPV	Positive predictive value
TIN	Testicular intraepithelial neoplasia
TSS	Testis-preserving surgery
US	Ultrasound
βhCG	Human chorionic gonadotropin
